# Fermentation-driven interactions of gut microbes with their environment

**DOI:** 10.1042/EBC20253057

**Published:** 2026-03-31

**Authors:** Alex Berretta, Clarissa Schwab

**Affiliations:** 1 Department of Biological and Chemical Engineering, Aarhus University, Aarhus, 8000, Denmark

**Keywords:** fermentation, gastrointestinal tract, microbiota, short chain carboxylic acids, short chain alcohols

## Abstract

The gut microbiome has gained a lot of attention in recent decades due to the multitude of interactions it has with the host. One of the main ways the microbiota communicates with the host is through the fermentation of dietary or host-derived nutrients. Fermentation of carbohydrates and amino acids yields structurally and compositionally different metabolites that have distinct functionality within the gut microbial community but also in the interaction with the host. The most abundant fermentation metabolites are the short-chain carboxylic acids acetate, butyrate, and propionate. While important contributions to host health have been attributed to these three, there are other compounds formed by fermentation whose relevance in the gut becomes increasingly recognized. In this essay, we will present how gut physiological properties relate to microbial fermentation capacity. We will introduce the diversity of fermentation pathways and relate functionality to the intrinsic properties of fermentation-derived metabolites. Finally, we will present strategies to restore disrupted fermentation activity.

## Introduction

The human gut microbiome encompasses the microorganisms inhabiting the gastrointestinal tract (GIT) together with the metabolites they produce [1]. This multifunctional community interacts with the host from birth [2]. After a dynamic development period during the first months of life, microbial community composition and enzymatic and metabolic activity stabilize with the introduction of solid food and remain fairly stable throughout adult life. Microbiota composition changes with aging and can be affected by the onset of GI diseases [3,4]. The microbiota and the host interact through the exchange of nutrients, which is a key aspect contributing to the health of the host. These interactions affect the maturation of the host immune system, the gut barrier function, and the resistance against invading pathogens.

One major biochemical process in the gut relevant to microbe-host interactions is fermentation. Fermentation is an anaerobic chemotrophic metabolism that does not require oxygen as an electron acceptor [5]. Energy is conserved through the formation of small metabolites, including short-chain carboxylic acids (SCCA) and short-chain alcohols (SCALC) that are released from the cells. Gases such as hydrogen sulfide and methane are also important byproducts [6]. Fermentation can also occur in aerobic conditions when substrate concentrations are high, e.g., during overflow metabolism. A switch towards fermentative rather than respiratory pathways can give a competitive advantage to specific members of complex gut communities [7].

**Figure 1:**
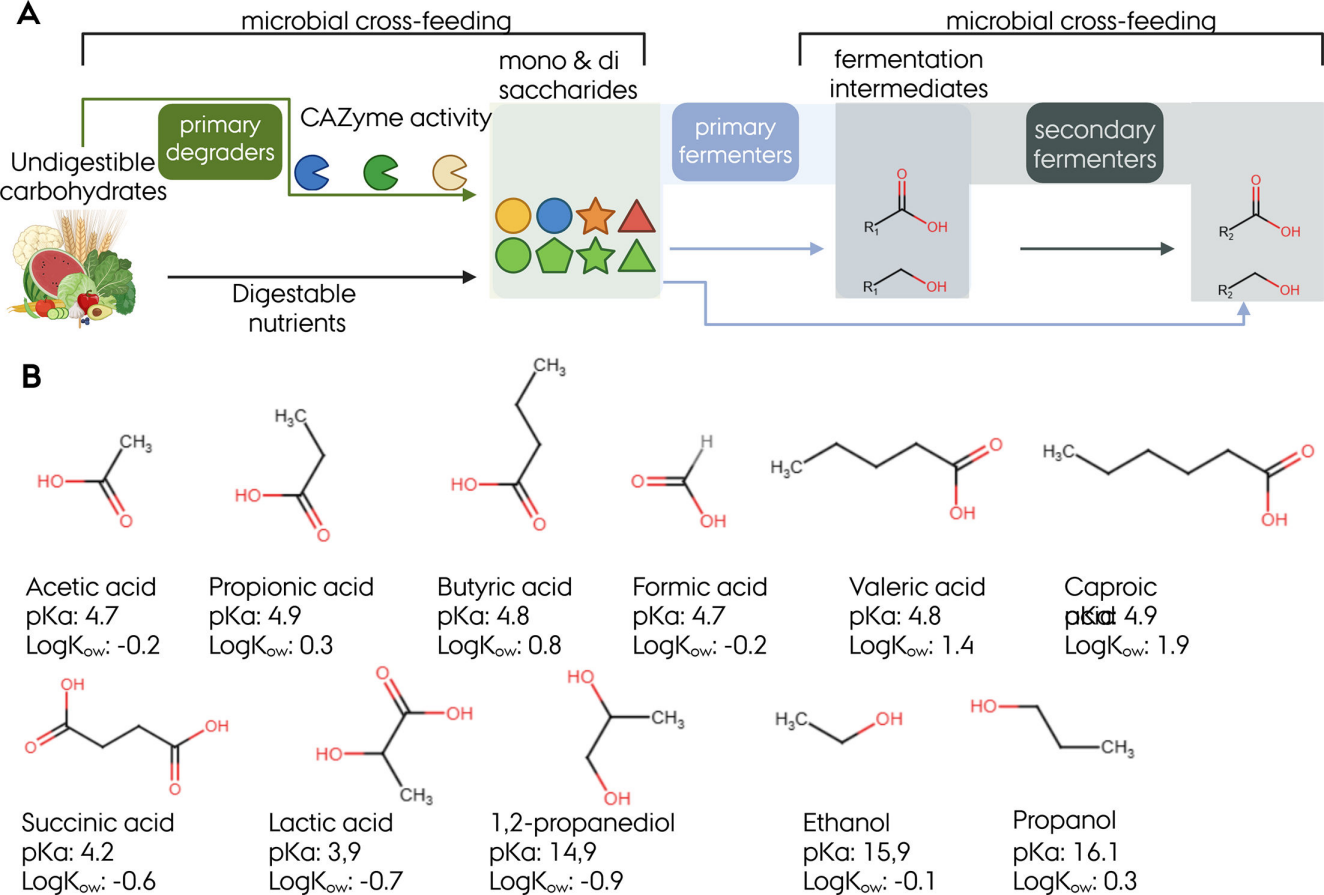
General microbial cross-feeding schemes and major fermentation metabolites. (**A**) Representation of microbial carbohydrate degradation and fermentation schemes. (**B**) Major SCCA and SCALC present in the human GIT with their chemical structure, pKa, and hydrophobicity coefficients. Functional groups are indicated in red. pKa and logK_ow_ were retrieved from PubChem. The figure was prepared with Biorender (license DV29J54W4M).

In the gut, the utilization of dietary nutrients is a successive and collaborative microbial activity with specialist microbial community members contributing to different trophic levels. The main fermentation substrates are carbohydrates. Specialist populations include primary degraders, primary fermenters, and secondary fermenters. Primary degraders release mono- and disaccharides from complex dietary polysaccharides (for example, resistant starch, β-glucan, arabinogalactan, xylan, fructans) or endogenous sources like mucin, through the activity of carbohydrate-active enzymes (CAZymes) (Figure 1A), which are utilized by primary fermenters [8,9]. Secondary fermenters feed on intermediate fermentation metabolites, including the SCCA lactate, formate, succinate, and acetate, and the SCALC ethanol, propanol, and 1,2-propanediol (1,2-PD) that are provided by the primary fermenters (Figure 1A) in a process called cross-feeding. The fermentation activities in the gut lead to the production of different SCCA and SCALC end products, which are either expelled via defecation or absorbed by colonic epithelial cells (Figure 1B). From a chemical perspective, SCCA and SCALC are weak acids (Figure 1B) that confer antimicrobial activity depending on environmental pH, compound structure, and hydrophobicity [10].

While the primary final metabolites are the SCCA acetate, propionate, and butyrate, intestinal fermentation yields a multitude of compositionally and structurally different compounds that play a role in interaction with the host but also in microbe-microbe communication.

Disturbances in intestinal fermentation activity have been linked to different disorders, highlighting the importance of a functioning intestinal fermentation microbiome. Yet, microbial communities and their contribution to intestinal fermentation differ along the GIT [11].

### The compartmentalization of the gut and its segmented fermentation activity

**Figure 2:**
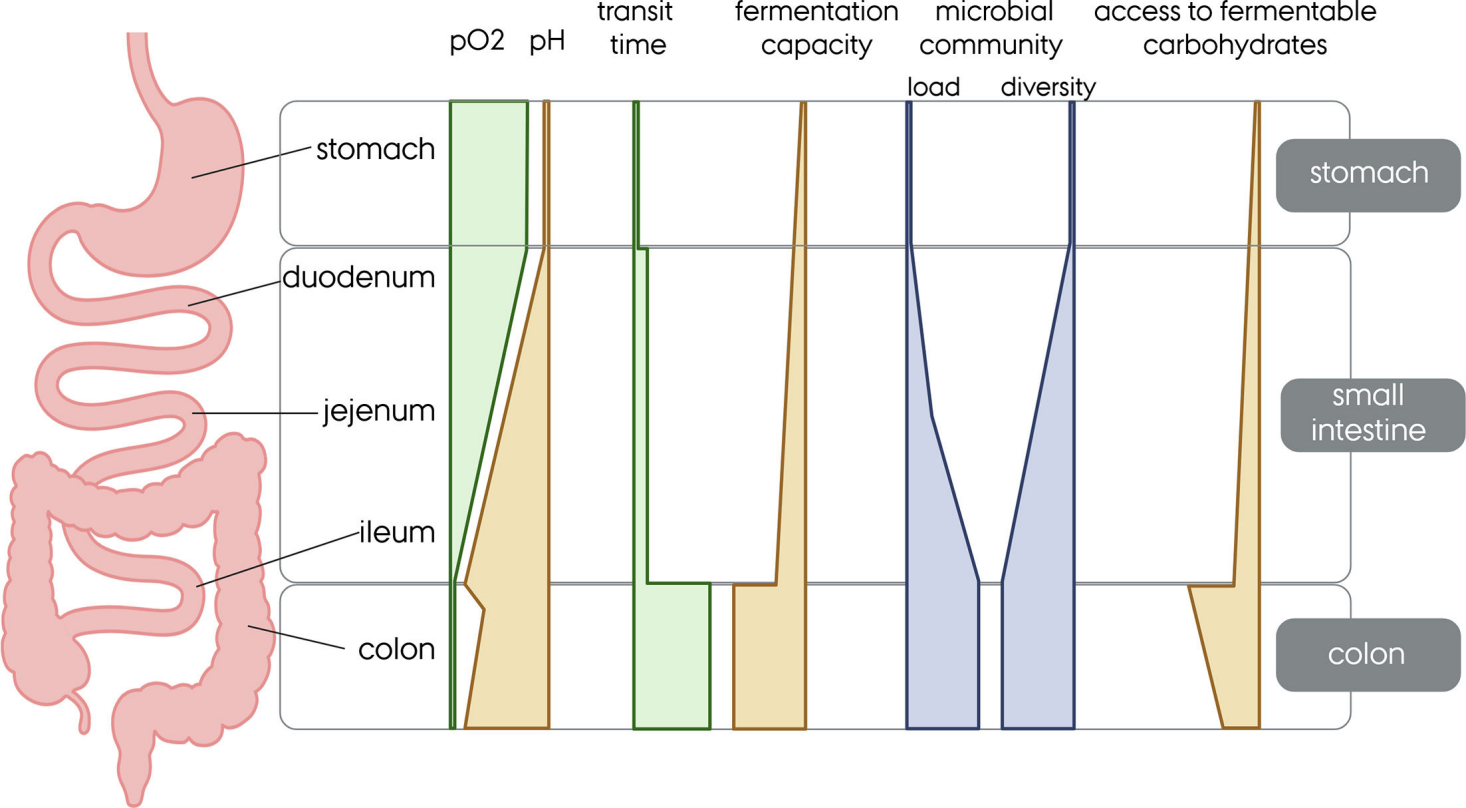
Schematic representation of different segments of the GIT and the impact on physical-chemical conditions and microbial populations. The figure was prepared with Biorender (license TL29J55DP3).

The human GIT is highly spatially compartmentalized (Figure 2), which allows for optimal digestion and absorption of most nutrients but also crucially affects microbial fermentation activity. Compartmentalization is the reason for the co-existence of multiple microbial communities with differences in taxonomic richness and functional (fermentation) potential. The GIT is divided into three main compartments: the stomach, the small intestine, and the large intestine (Figure 2).

The stomach is fundamental to the chemical and mechanical degradation of food. Few microbes, including the pathogen *Helicobacter pylori,* are able to colonize the stomach because of the acidic pH and the presence of antimicrobial peptides [12]. Additionally, recent studies involving healthy individuals and subjects with disorders in the upper GIT suggested the presence of transcriptionally active microbes [13,14]. The contribution of the stomach to overall fermentation activity in the GIT is likely low [15].

The small intestine is the main compartment for nutrient absorption by the host and harbors a heterogeneous microbiota throughout its length [16]. The pH ranges from around 6 in the duodenum to around 7.5 in the distal ileum [17], and there are also higher levels of oxygen (10 to 60 mmHg) compared with the colon [18]. Critical factors influencing the composition and fermentation activity of the small intestinal bacterial community are a short transit time (2 to 5 h) [19], competition with the host for nutrients, and the release of antimicrobial peptides from the host epithelium [20]. These harsh conditions allow for a low-abundant bacteria population of 10^3^ to 10^8^ cells/ml from the proximal to the distal part of the small intestine [21]. Microbes that grow fast have a competitive advantage in metabolizing simple nutrients. For example, lactic acid bacteria such as *Streptococcus* sp. utilize mono- and disaccharides to produce lactate [22]. Only low levels of fermentation metabolites have been recovered in the small intestine [23], pointing to limited fermentation capacity. While more complex carbohydrates are available, the potential for degradation is limited due to time constraints and a restricted repertoire of CAZymes of the small intestine microbiota [19,24]. A significant part of the ingested carbohydrates traverses the small intestine untouched; these undigestible carbohydrates become available to the colonic microbiota.

The large intestine, also referred to as the colon, is the primary residence site of our gut microbiota. It harbors around 10^11^ bacterial cells per ml, including populations of primary degraders and primary and secondary fermenters (Figure 1A) [21]. In healthy conditions, the colon provides an anoxic environment, with less than 5 mmHg of oxygen pressure and a pH that ranges from 6.5 to 7, which are optimal conditions for anaerobic microbes [17]. Colonic transit is highly individual (10–59 h) but still longer than in the small intestine [25–28], allowing for extensive carbohydrate degradation and fermentation. While little data is available from humans, studies in monogastric animals indicated that SCCA levels were up to 10-fold higher in the colon than in the small intestine, pointing to the colon as the primary fermentation site in the GIT [15] [1,2]. In the colon, propionate and butyrate have a role as energy sources for colonocytes [29], and butyrate is a key regulator of mucin secretion and inflammation status [30–32], highlighting the tight relationship of fermentation metabolites and host health.

### The big three produced in the colon: acetate, propionate, and butyrate

**Figure 3:**
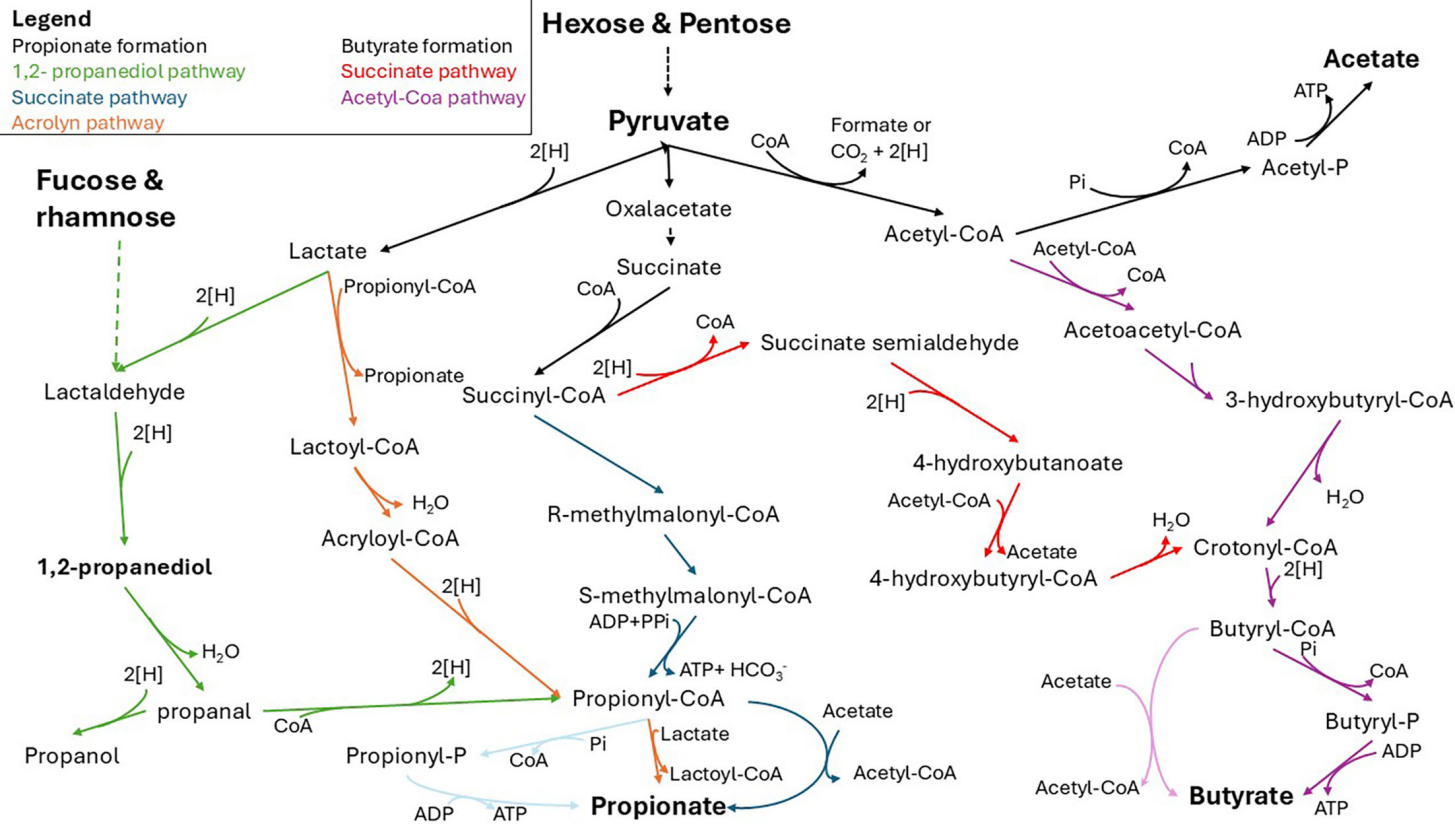
Major fermentative pathways to produce the main SCCA and SCALC assembled based on [35,36]. Distinctive pathways are highlighted by colored arrows.

Colonic microbial fermentation systems concurrently yield SCCA and SCALC, whose levels are often measured in feces as a proxy. Fecal levels are likely affected by GI transit time, stool frequency, volume of stool output, and adsorption capacity. Frequently used analytic systems are gas, liquid, or ion chromatography based with refractive index, mass spectroscopy, or suppressed conductivity detection [33,34]. Among the fermentation metabolites, SCCA, which possess at least one carboxylic group (Figure 1B), are major fermentation metabolites of gut microbes. Due to a near-neutral pH of the colon environment, 90-99% of SCCA are present as anions rather than as free acids, which limits their antimicrobial activity [10]. Acetate production is the most widespread across gut microbes, while butyrate and propionate production are less common and associated with microbial specialists [35]. Accordingly, acetate levels are usually three times higher than butyrate and propionate [15]. Acetate is mainly produced through the decarboxylation of pyruvate via acetyl-CoA (Figure 3). It can also be derived from acetogenesis, through the reduction of CO_2_ and the utilization of H_2_ or formate as electron donors [37]. The formation of butyrate from the fermentation of carbohydrates also relies on the production of acetyl-CoA (Figure 3) [36]. Acetyl-CoA is then converted to the intermediate butyryl-CoA in a four-step process. The final reaction to butyrate is either catalyzed by butyryl-CoA:acetate CoA transferase or butyrate kinase [3–9,38,39]. Butyrate can also be produced from succinate, but few microbes have been identified with this capacity to date [40].

Propionate can be formed through the succinate, acrylate, and the 1,2-PD pathways [36,41] (Figure 3). The succinate pathway [10,11] has been predicted to contribute the most to intestinal propionate formation [41]. Succinate is converted to methylmalonyl-CoA via an intramolecular hydroxyl group transfer and the addition of CoA. The decarboxylation of methylmalonyl-CoA leads to the formation of propionyl-CoA, which is transformed into propionate by a CoA-transferase or a kinase similar to the reactions from butyryl-CoA to butyrate.

The acrylate pathway begins with lactate and yields propionyl-CoA, with acryloyl-CoA as an intermediate. The 1,2-PD pathway uses rhamnose and fucose as substrates for propionate formation. The metabolism of these deoxyhexoses yields the diol 1,2-PD, which is further metabolized to propionate and the SCALC propanol (Figure 3). This pathway is performed in a proteinaceous microcompartment, due to the production of propanal as an intermediate, which is highly volatile and toxic to the cell [42].

In fecal samples from healthy adults, fermentation intermediates such as succinate, lactate, and 1,2-PD are typically not detected or are present only at low levels, indicating an intact fermentation system.

Disruptions in this complex fermentation network can result in imbalances of fecal SCCA profiles. For example, in addition to other changes in metabolite profiles, a lower abundance of butyrate producers and fecal butyrate was observed in patients with inflammatory bowel disease [43]. This may be due to the alteration of oxygen availability in the colon, which causes a shift in microbiota towards genera more commonly associated with the oral microbiota and a disruption of the normal metabolite production [44,45].

### Why not longer? Valeric and caproic acid are also produced by intestinal microbiota

**Figure 4:**
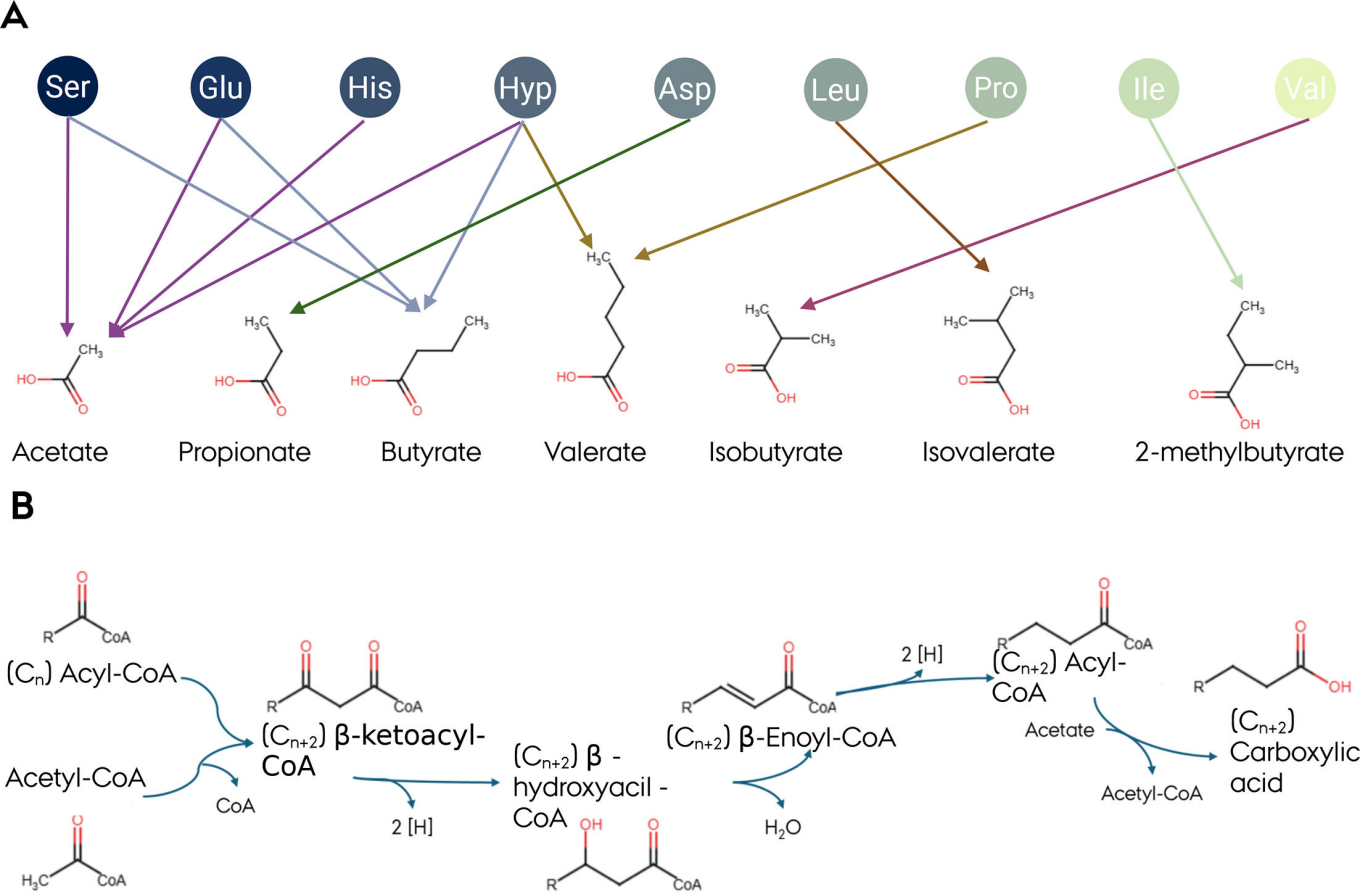
Schematic representation of amino acid fermentation and BCCA formation. (**A**) Amino acids fermentation to SCCA based on Stickland reactions; (**B**) Example of a chain elongation pathway to produce carboxylic acid. The pathway depicted is a reverse beta oxidation pathway, with the R group representing an aliphatic chain. This pathway can lead to the formation of caproate and valerate if the initial metabolite is butyryl-CoA or propionyl-CoA, respectively [47,48]. The figure was prepared with Biorender (license FX29J55QTX).

SCCA with a backbone of a maximum of four carbons has been most investigated in the context of human gut microbiota fermentation activity. However, SCCA with five or six carbon residues, *i.e*., valerate and caproate, are also metabolites produced by microbes in our GIT. Both molecules are formed through chain elongation of propionate (also from lactate) and butyrate with acetyl-CoA in anaerobic conditions [46–48] (Figure 4B). For example, Huertas Diaz et al. recently showed that the genus *Megasphaera* contributes to valerate formation in the human GIT, especially after the consumption of fermented dairy containing lactate [49]. Valerate can be produced by a Stickland reaction from proline and hydroxyproline [50], but no specific microbial taxon has been associated with this pathway in the gut [51].

Due to the structural similarity of valerate and caproate with propionate and butyrate (Figure 1B), the pKa of these compounds is similar. However, valerate and caproate are more hydrophobic and stronger antimicrobials than their shorter counterparts, with some activity even at near neutral pH [52]. High levels of these SCCA might not be favorable in the GIT. Nonetheless, there is increasing evidence that the presence of valerate relates to health status. Restoring valerate levels improved health prognosis for patients with *Clostridoides difficile* infections [53]. Fecal valerate levels were negatively correlated with disease severity of ulcerative colitis in humans [54], similar as it was reported for butyrate. Whether there is a valerate-specific effect or an indication of a generally modified fermentation activity remains to be investigated.

Much less is known about caproate, which cannot be detected with analytical systems that are routinely used for metabolite analysis such as liquid chromatography with refractive index detector (personal observation). Nonetheless, caproate was detected in the colon and in fecal samples of monogastric animals at levels around 10-fold lower than valerate (0.1 to 0.4 vs 1 to 4 µmol/g_feces_) [55], with some key bacterial species, e.g., *M. elsdenii*, reported to produce caproate [46]. Levels of caproate were several-fold less than the SCCA acetate, propionate, and butyrate.

Together, these observations indicate that the formation and roles of valerate and caproate remain understudied in gut fermentation systems.

### Contribution of amino acid metabolism to carboxylic acid formation

In addition to carbohydrate fermentation, acetate, butyrate, and propionate can be produced through the metabolism of specific amino acids. These processes are not preferred when other carbon sources are available [50]. The fermentation of certain amino acids can also lead to the formation of branched-chain carboxylic acids (BCCA). The main BCCA are isobutyrate (from valine), isovalerate (from leucine), and 2-methylbutyrate (from isoleucine) [56] (Figure 4A). Formation of BCCA is most probably performed through a Stickland reaction, which enables bacteria to generate ATP by coupling the oxidation and reduction of amino acids [57]. BCCA has not gained the same attention as SCCA, possibly due to low concentrations in fecal samples. In fact, BCCA usually reaches cumulative levels lower than 5 mM, accounting for less than 10% of the total fecal SCCA and BCCA [58].

Higher levels of BCCA have been related to a high-protein diet in an *in vitro* gut model and in piglets [59,60]; whether a similar relationship exists in humans remains to be investigated. At the same time, concentrations and proportions of BCCA were significantly lower in healthy individuals than in patients with certain metabolic disorders [61,62]. In addition, molar proportions of fecal BCCA isovalerate and isobutyrate increased with age [58], highlighting that further investigations are needed to determine whether there is a health or disease-related functionality of BCCA in the gut.

### Let’s not forget the alcohols

In addition to SCCA and BCCA, alcohols are produced by fermentation activity. 1,2-PD, which is also known as propylene glycol, is used in technical applications such as antifreeze, emulsifier and anticaking agent. In the infant gut, 1,2-PD is produced during the degradation and fermentation of fucosylated human milk oligosaccharides and accumulates at high levels in feces [63]. In contrast, 1,2-PD is considered a fermentation intermediate in adults and has been rarely found in fecal samples. Usually, 1,2-PD is further metabolized to propionate and propanol by the 1,2-propanediol pathway (Figure 3). Studies that explicitly mention the detection of propanol in feces are rare [33,63].

Another alcohol, ethanol, is part of the social life of almost all adults around the world. However, we fail to consider that ethanol is produced by intestinal bacteria and yeast as well. Endogenous ethanol is described in a range between 0 and 3 mg/dl in peripheral circulation in adults [64]. The concentration can increase after the consumption of alcohol-free meals, pointing to the contribution of microbial fermentation and endogenous alcohol formation. Endogenous ethanol fermentation is mainly produced by mixed acid fermentation or the 2,3-butanediol pathway [65]. In both pathways, the production of ethanol maintains the redox balance [5].

The mixed acid fermentation pathway leads to the formation of different metabolites, e.g., formate, lactate, citrate, and succinate, depending on environmental stress conditions, redox balance, and nutrient availability [5]. The 2,3-butanediol pathway, which is utilized by *Klebsiella spp*. and members of the *Enterobacteriaceae* family, supports pH buffer capacity to reduce cellular death from acidification [66]. In normal conditions, endogenous ethanol is detoxified by the liver; ethanol accumulation can cause liver malfunctioning [67] or Auto Brewery Syndrome (ABS). In ABS, endogenous ethanol concentrations are so high that patients show symptoms of alcohol intoxication [68]. ABS has been often associated with yeast populations colonizing the GIT of patients. *Klebsiella pneumoniae* is one of the few bacterial species linked to ABS [69]. In addition to ethanol, levels of 2,3-butanediol significantly increase with the onset of an alcohol fermentation episode of ABS [70].

The relationship between endogenous alcohols and disease states calls for further investigations and possible implications of other SCALC.

### Strategies to restore disturbed fermentation systems

Several links of disruptions in fermentation metabolism and health call for strategies to enable the restoration of intestinal fermentation capacity. However, there is no panacea: due to the diversity of disturbances that affect taxonomically different producer strains that employ various fermentation pathways, tailored remedies must be used for each problem.

The most straightforward approach to addressing the intestinal lack of specific SCCA is the reintroduction through oral supplementation. Oral uptake of different forms of butyrate (Na-butyrate, tributyrin) has been tested for some inflammation-related conditions, but the effects were only transient [71,72]. There is also limited consumer tolerance because butyrate and other SCCA have specific organoleptic properties. To circumvent issues with taste or absorption in the small intestine, SCCA can also be administered rectally, and multiple studies report at least a transient recovery of gut functionality [73,74]. However, almost all do not mention whether these treatments led to a recovery of the healthy fermentation network.

Another way to replenish specific fermentation metabolites is through dietary interventions, i.e., through the introduction of prebiotics. Prebiotics are defined as: ‘a substrate that is selectively utilized by host microorganisms conferring a health benefit’ [75]. *In vitro* in gut models, prebiotics such as inulin led to higher butyrate levels due to microbial cross-feeding [76]. Meta-analyses highlighted conflicting results regarding prebiotic consumption and SCCA production *in vivo* [77,78], possibly due to differences in methodologies, study duration, and prebiotics used, as well as the responder status of the host.

More forceful dietary interventions, *i.e*. the adherence to a completely new diet scheme for a period of time, can have an impact on the GIT fermentation activity. A long-term Mediterranean diet intervention [79,80] led to an increase in fecal butyrate and acetate, with a concurrent reduction in inflammation. Almost counterintuitively, intermittent fasting, which alternates periods of complete fasting and mealtimes, also affected intestinal fermentation activity. A study on diet-induced obese mice showed that intermittent fasting increased fecal SCCA levels even with a hypercaloric diet [81]. There are still too few studies in humans to evaluate the overall efficacy of such an approach [82].

As an alternative to dietary modulation, the introduction of defined microbes is another strategy to restore disrupted fermentation systems. Probiotics are defined as *live microorganisms which, when administered in adequate amounts, confer a health benefit on the host* [83]. Evidence of probiotic use can be found as early as ancient Rome [84]. While the concept of probiotics remains a matter of debate, ‘classic probiotics’ such as *Lactobacillaceae* and B*ifidobacterium spp*. produce lactate and acetate [85], which can contribute to microbial cross-feeding activities. In contrast to probiotics, live microbial biotherapeutics or next-generation probiotics target a sick population and are microbes supplemented for specific therapeutic reasons. Such biotherapeutics were already suggested to modify intestinal fermentation patterns. For example, microbial strains overexpressing alcohol and acetaldehyde dehydrogenases restored gut barrier function in mice with ethanol-induced liver disease. Strains of the biotherapeutic candidate *Faecalibacterium* have been suggested for several applications partly because of the ability to produce butyrate [86,87]. The administration of already established communities, most commonly taken from fecal material of healthy donors, represents another strategy to reinstall a functional fermentation network [88]. Fecal microbial transplant (FMT) performed on a mouse model for colitis and on a UK cohort with active ulcerative colitis indicated that FMT can improve SCCA levels through reintroduction of key SCCA producers [89,90]. However, the success of FMT might relate to the levels of disturbance and remaining butyrate producers, as some patients with recurrent *Clostridium difficile* infection had a depletion in common butyrate producers both before and after the treatment [91]. Individuality of each person’s gut microbiota can also hinder the efficacy of probiotics, biotherapeutics, and FMT, calling for a more ‘person-specific’ treatment [92].

These results underline even more the importance of a functioning fermentation network for human health.

## Conclusion

The fermentation network in our gut, and especially in the colon, is undoubtedly a key component in the interactions within the microbes and between the host and its intestinal microbial community. Currently, our main knowledge is limited to butyrate, propionate, and acetate, and we are far from having a complete overview of the functions of other fermentation metabolites in adults, as well as other stages of life. Even for propionate and butyrate, new interactions continue to be discovered. For example, butyrate and propionate have been implicated in histone modification [93,94], but the function of such modifications is still not well understood. With deeper insight into the mechanisms of action of fermentation metabolites, targeted strategies can be developed to rationally modulate the fermentation microbiome, particularly during periods of disruption.

**Summary points**

- Fermentation of dietary nutrients is an essential way for the gut microbiota to gain energy and to interact within itself and with the host.
- There are multiple SCCA and SCALC produced by fermentation pathway, and only a few have been thoroughly studied.
- Multiple disorders have been linked with a disruption of the fermentative networks within our gut, and different approaches aim to restore the equilibrium.

## Open Access

Open access for this article was enabled by the participation of Aarhus Universitet in an all-inclusive Read & Publish agreement with Portland Press and the Biochemical Society under an agreement with the Royal Danish Library.

## Abbreviations

GIT: gastrointestinal track
1,2-PD: 1,2-propanediol
SCALC: short-chain alcohols
SCCA: short-chain carboxylic acids
